# Cancer

**Published:** 1906-02-10

**Authors:** 


### Progress in Medicine and Surgery.

### CANCER.

**Parasitic Theory.**—The idea that cancer may be due to some parasite has great attractions for many, especially as, if proved, it seems to be the first step towards general curative measures, far exceeding in their scope and certainty anything which we now possess. Von Leyden [1] sums up the general evidence as follows: Cancer is common only among domesticated animals, and those most frequently attacked are those living near pathological institutes and laboratories. It has a wide distribution, but is not found in antarctic latitudes. It was unknown among African negroes, but it is common enough among imported American negroes. It is on the increase in large towns, and occasionally occurs in small epidemics. The parts most frequently attacked are those accessible from without. Certain chemical differences between cancer cells and ordinary tissue cells, as well as transplantation experiments with the former complete the evidence. Von Leyden does not accept the bacterial theories, but thinks he has observed the parasite in certain cell inclusions, probably protozoa. He does not hold that the failure to cultivate these on artificial media invalidates their claim to be regarded as the specific cause of cancer, as they are essentially intracellular organisms. On the other hand, Israel [2] has pointed out that in no known instance do cells containing a parasite divide and multiply—either the cell or the parasite dies; while both hold that though possibly true, the parasite theory has never been yet proved, no one having succeeded in producing cancer experimentally without transplanting the actual cells, or having demonstrated parasites characteristic of each form of malignant tumour. Hansemann [3] also considers that all the organisms hitherto described fall short of what is necessary to prove their etiological significance. He does not attach any importance to the reported cases of individual infection or epidemic occurrence in a condition so widely spread as cancer. Oldhausen and Benda [4] support the parasitic theory as the one which explains best most of the observed facts.

**Doyen's Microbe.**—This organism, which has been called the Micrococcus neoformans, has been credited by Doyen [5] with the production of malignant growths of all kinds. He thinks that it exists in all tumours, which arise from its irritant action on the tissue cells, and that the supervention of malignancy depends upon a special reaction between the microbe and the cells of the tumour. He can stain and cultivate the organism, and increase or decrease its virulence by special means. He has found it in connection with various new growths, in affected glands, and in the lymphatics in a pre-cancerous condition. In six animals inoculated with cultures tumours developed after an interval of some months. Doyen has produced a "vaccine" or attenuated culture which he has injected into horses; the serum of these horses has been used as a therapeutic agent. In cancerous subjects it is said to produce a reaction analogous to that of tuberculin. Doyen has used this serum, either with or without surgical treatment, in 242 cases, of which 42 are considered cured, others are either improved, lost sight of, or still under observation, and 128 have not been benefited. He does not claim any success in the last stages of cancer, but thinks the serum may be used (1) prophylactically, (2) as an absolute cure, either with or without operation, to ensure nonrecurrence (immunity), (3) to render unoperable cases operable by reducing the size and extent of the growth. Metchnikoff has confirmed the existence of this microbe in cancers, but cannot state positively that it is the cause of their growth. Weinberg, one of his assistants, could not consider the lesions experimentally produced in animals by Doyen as malignant, but thought them purely inflammatory, a conclusion at which Cornil and other investigators of the Société Anatomique independently arrived. Weinberg moreover states that the alleged causal microbe plays in reality only a secondary part, and that the agglutinating reaction which Doyen demonstrated was not convincing. Metchnikoff himself, who endorses the views of his assistant, thinks the therapeutic successes of Doyen's serum are similar to those of antistreptococcal serum in some cases of phthisis.[6] Working on similar lines, Gaylord, Clowes, and Baeslack[7] have obtained from mice who appeared to possess some natural immunity, a serum for which they claim active immunising powers. As far as their experiments go, they are inclined to believe that the active agent is not a cytolysin; the epithelial cells of the cancer in the animals treated undergo simple atrophy while the connective tissue increases to a large extent. They apparently think, therefore, that the immune body acts on some parasite or other agent which causes the cancer. These experiments, interesting as they are, are far from carrying conviction at their present stage. Even supposing that Doyen's results were confirmed, which hitherto they have not been, by other observers, it does not appear that his serum is a "cure" for cancer in any greater degree that Koch's old tuberculin was a "cure" for phthisis. In fact, his claims for his serum amount to much the same as is now claimed for various antituberculous vaccines and sera. It is said to be a useful adjuvant, to cause the disappearance of tumours in favourable cases, and to be useless in the later stages of the disease. The main objection to it seems to be that there is no proof that his micrococcus neoformans is the cause of malignant growth; and thus even less than the antituberculous sera can it claim to contain active antibodies to the disease.

**Plimmer's Bodies.**—These curious bodies, which were described some years ago have recently been investigated by Farmer, Moore, and Walker.[8] They find that these bodies and the changes which they undergo are closely comparable to the appearances seen in the cells during the production of male sexual elements in man and other animals. This observation affords a striking corollary of the resemblance between the methods of nuclear division previously described in reproductive tissue and malignant growths, but the authors adhere to the opinion they formerly expressed, that the two tissues are far from being identical though resembling each other in important particulars. Their present observation is not explained, but it is suggested that it may be an instance of reversion to or retention of some ancestral characteristics.

**Germ Centres and Carcinoma.**—A short paper by Cleland[9] illustrates one of the difficulties among the many which are involved in the investigation of cancer. There exist in normal lymph glands certain areas known as "germ centres," which differ from the ordinary lymphatic tissue. They are composed of pale cells having more protoplasm and a larger vesicular nucleus than ordinary lymphocytes, and they stain less deeply. They are not cut off from the ordinary lymphoid tissue by any hard and fast line, but rapidly merge into it, mitotic figures being frequent in the nuclei of the cells in the intermediate areas. Under the stimulus of chronic irritation, however, the cells become larger, and mitotic figures more frequent, as if compensatory hypertrophy were going on: at the same time they become more distinctly divided from the tissue in which they lie. They thus closely resemble secondary carcinomatous deposits, the main distinctions being that carcinomatous cells are not so uniform in size or so regularly arranged, and are often separated off by a very fine band of connective tissue. In hypertrophied germ centres under a high power gradations into normal tissue can usually be made out, though in some cases the distinction may be very difficult to make, especially under a lower magnification.

**Dissemination of Cancer.**—A most important contribution to this subject is found in Handley's Hunterian lectures for 1905.[10] The statistical part of his lectures is based on a very large number of cases, and the histological part on some careful and original observations. His method has been to cut out long strips of tissue radiating from the primary growth (his observations are on cancer of the breast), then to cut these transversely into pieces suitable for microscopical examination and to number and section them. By this very laborious process, involving the preparation and examination of some 500 sections, he has been able to trace systematically the path by which cancer spreads in the tissues of the body, and to draw some ingenious and convincing deductions from his observations. There are three conceivable methods by which cancer cells may reach distant organs; they may be swept into the lymph-stream, or grow directly along the lymph channels, and they may reach the blood-stream and become lodged as emboli in the viscera. The latter possibility is first discussed by the lecturer, who shows that it can only very rarely occur. In the first place, the peculiar distribution of metastatic growths is not what is to be expected from an impartial process like embolism; then it has been shown that cancer cells, if they do form emboli, are usually rapidly ensheathed by organised blood clot, and become unable to grow when thus deprived of nutritive matter. Moreover, it has also been shown that the blood itself possesses a high specific resistance to cancer cells. As to the lymph-stream, Handley remarks that when the cancer cells have reached the lymph plexus in the deep fascia beneath the breast, they may (1) reach the axillary glands, and so reach the blood-stream, when they are rapidly destroyed, or (2) reach the periphery of the lymphatic plexus by a reflux from the glands, where they will be stopped as by a filter, owing to the fineness of the capillary lymph vessels. He concludes that the only remaining way in which cancer cells can spread is by actual growth along the lymph vessels, just as bacteria, if left on a porcelain filter, will in time grow through it, though they cannot be forced through by direct pressure. This process of growth is called by Handley "permeation," and is shown by his histological sections to occur radially from the primary growth; he is able thus to account rationally for the various metastases which commonly occur. Thus, he shows that the humerus is generally attacked about its middle third, and the femur just at or below the great trochanter, that is where the bones are nearest to the surface of the body and the lymphatics of the deep fascia, along which the cells have grown. Those operators, moreover, who remove the deep fascia widely are shown statistically to obtain better results than those who merely remove a wide area of skin. The abdominal and thoracic invasions by secondary growths are shown to be distinct events in most cases, the former occurring much earlier than the latter. The path by which the cancer cells gain access to the peritoneum is shown microscopically to be via the epigastric angle, the lymphatics there joining those leading into the body cavity. Once inside the peritoneum the cancer cells often gravitate into the pelvis, causing secondary growths there, or they may invade the liver by the lymphatic communications. The thorax is invaded much later, and it is only in late cases that the peritoneum is invaded secondarily by extension through the diaphragm. Handley shows that there is reason to believe that pleural adhesions afford a certain protection to the pleural cavity, if sufficient to obliterate it.

**The Practical Conclusions** are that the modern operation affords sufficient protection for the thorax, but not for the abdomen and the parietal structures of the body, because few operators have realised the importance of a widespread removal of the deep fascia with its lymphatics, especially towards the epigastric angle, when the chains of cancer cells grow over into the abdominal cavity. He thinks that the deep fascia should be removed much more widely than the skin and more superficial structures, and that possibly there might be some advantage in artificially obliterating the pleural cavity on the same side.

**Pathological Point.**—It is interesting to note, from a pathological point of view, the importance which Handley attaches to recent work which shows how difficult it is for cancer cells to multiply in the blood-stream, partly owing to the destructive or protective properties of the blood. Chorion-epithelioma is, as he points out, the only form of cancer which is at all commonly spread by the blood-stream, and in this, spontaneous disappearance of metastatic growths is also most common. This should be considered in relation to the attempts, recently referred to in these columns, to produce anti-cancerous sera. It supports the view that such sera are scientifically possible, but inasmuch as cancers are so seldom disseminated by the blood, it limits the practical application of sera, especially in cases where metastatic growths are widely spread.

[1] Med. Rec., May 6, 1905, p. 699. [2] Ibid., p. 700. [3] Ibid. [4] Ibid. [5] Edin. Med. Jour., April, 1905, p. 373. [6] Lancet, April 8, 1905, p. 955. [7] Brit. Med. Jour., March 11, 1905, p. 542. [8] Ibid., June 10, 1905, p. 1277. [9] Lancet, Sept. 16, 1905, p. 820. [10] Ibid., April 8, 1905 p. 909; April 15, 1905, p. 983; April 22, 1905, p. 1047.

*(To be continued.)*

